# Trends and future of maternal and child health in Bangladesh

**DOI:** 10.1371/journal.pone.0211875

**Published:** 2019-03-15

**Authors:** Sultana Rajia, Md. Sabiruzzaman, Md. Kamrul Islam, Md. Golam Hossain, Pete E. Lestrel

**Affiliations:** 1 Department of Statistics, Rajshahi University, Rajshahi, Bangladesh; 2 Professor Emeritus, Sections of Orthodontics and Oral Biology, School of Dentistry, University of California, United States of America; The University of Warwick, UNITED KINGDOM

## Abstract

**Background:**

Maternal and child health is one of the most important issues in a developing country like Bangladesh. This study evaluates the trends in maternal and child health indicators of Bangladesh.

**Methods:**

The secondary data used in this study was extracted from the World Bank Dataset. The selected indicators were maternal mortality ratio (MMR), under-five children mortality and neonatal mortality rate, and prevalence of stunting and wasting of under-five children. Trend analysis technique and ARIMA forecasting models were used in this study to find currents trend and predict the future of selected indicators.

**Results:**

This study revealed clear evidence that neonatal, under-five child and maternal mortality in Bangladesh had been gradually decreasing during the last two and half decades. The decreasing rate of these indicators suggests that it should be possible to achieve the national target of sustainable development goals (SDGs) of Bangladesh by 2021. While, it was observed that the prevalence of underweight, stunting and wasting among under-five children was still high, these indicators have been slowly decreasing over time. The decreasing rate of these indicators displayed that without guided measures, the Bangladesh government would not be able to achieve the target goal of child malnutrition by 2021 under SDG-2.2.

**Conclusion:**

It is recommended that the government, as well as non-government health organizations (NGOs), and other policy makers should provide programs that are effective so that the national target goals can be achieved by the year 2030. Consequently, our findings should assist in the achievement of the national goals in Bangladesh regarding these health issues.

## Introduction

Bangladesh is a developing country in South Asia with a population of more than 142 million, a poverty level of 33% and also having lots of health related issues [1]. The greatest asset for Bangladesh is its people, especially its population of children. A wide-ranging health infrastructure in the public and private sectors has been established through a number of reforms after independence of Bangladesh in 1971.

The SDG-3 states “*To ensure healthy lives and promote well-being for all at all ages*”. The success of SDG 3 depends on progress in other SDGs—e.g. poverty reduction, nutrition, quality education, gender equality, clean water and sanitation, sustainable energy and safer cities. To achieve these health goals, a number of targets have been set. Multiple of these targets of SDGs are related to maternal, newborn and child health [2,3].

The maternal mortality ratio (MMR) per 100000 live births was estimated at 216 globally in 2015. The global MMR decreased by 44% during the Millennium Development Goal (MDG) era [4–5]. The MDG-5 target was to reduce the maternal mortality ratio from 574 to 143 deaths per 100,000 live births by 2015 in Bangladesh [6–7]. There has been a significant decline in the MMR rates; however, the trajectory is not sufficient to meet the targets. Skilled birth attendant is a critical intervention for reduction in maternal and newborn outcomes. The coverage of skilled attendance at birth was estimated to have reached 73% in 2013 worldwide. A major portion of children in the WHO African Region and WHO South-East Asia Region (more than 40% of births) are born without skilled health personnel [8].

In 2015, global under-five mortality rate was 42.5 per 1000 live births. Child mortality is highest in sub-Saharan Africa followed by South-East Asia. The annual rate of reduction in under-five mortality was 3.9% between 2000 and 2015 [9]. Bangladesh has experienced a significant reduction of child mortality over the past decades [10] which helped achieve the MDG-4 target. But the mortality among the under-5 children must be further reduced to achieve the Sustainable Development Goal (SDG) target. At this stage, it is very important to explore the trend of under-5 mortality to reduce the vulnerability of child’s survival.

Neonatal mortality is an influential part of overall child mortality. A specific target of 12 neonatal deaths per 1000 live births in 2030 was included in the SDG. There was a 3.1% decline in neonatal deaths between 2000 and 2015, and this rate of improvement would need to be maintained for achieving the child mortality target [11]. Neonatal mortality rate of Bangladesh fell gradually from 63.4 deaths per 1,000 live births in 1990 to 23.3 deaths per 1,000 live births in 2015 [3].

The indicators for SDG Target 2.2 on ending all forms of malnutrition are focused on stunting, wasting and overweight among children under 5 years of age. Almost one in four children under 5 years of age (23%, or 156 million children) were affected by stunting in 2015 worldwide. There were 50 million wasting affected children under 5 years of age (around 7%) globally in 2015 [3]. Prevalence of stunting, height for age (% of children under 5) in Bangladesh was 36.40 in 2014. Its highest value over the past 31 years was 76.70 in 1991, while its lowest value was 36.40 in 2014. Prevalence of wasting (% of children under 5) in Bangladesh was reported at 14.3% in 2014 [12].

Improving the well-being of mothers, infants, and children is an important public health goal for the world. Ensure sound health of next generation is one of the key challenges for public health care system of a country. To assess the achievement and future trend the public health sector should be monitored over time. Due to its importance in identifying health status of a country, trend analysis as well as forecasting of health indicators is addressed by a number of academics [13–22]. Although a number of cross-sectional studies are available [1, 6, 7, 23, 24], a limited number of studies are found on time series properties of public health of Bangladesh that summarize the history and subsequently predict the future [10,18].

The aim of this study is to evaluate the trends of maternal and child health indicators included in SDG of Bangladesh and predict for the national target-2021 taken by Directorate of Health, Bangladesh. This study will focus on the improvement in SDG targets-2030. Health indicators included in the study are Maternal mortality ratio (MMR), Under-five mortality rate, Neonatal mortality rate, Prevalence of stunting and prevalence of wasting which strongly indicate the maternal and child health status.

### Data and methods

This study is primarily based on a secondary data. The yearly dataset used in this study is freely available from the World Bank Data archive of Health, Nutrition and Population Data and Statistics category spanned from 1990 to 2014 (www.data.worldbank.org/topic/health). Missing data are estimated with a simple moving average technique. The data set used in this study is given in S1 Table.

The methodology involved trend analysis techniques to observe the trends in selected maternal and child health indicators of Bangladesh and ARIMA forecasting to predict the future scenario of these health indicators. Data analysis is done in R environment.

Trend analysis of time ordered observations using traditional regression model is not appropriate, since the errors of the regression line in such a case are typically autocorrelated. Presence of autocorrelation results sub-optimal estimate of the trend with incorrect estimate of standard error. This study utilizes ARIMA model to errors of the trend equation for treating autocorrelation problem and making prediction as well. The ARIMA model, introduced and popularized by econometricians, is an indispensable tool for modeling and forecasting sequence of observations in time [13–15].

Because of its flexibility, a polynomial trend model is implemented to represent public health indicator:

$$Y_{t}=\alpha +\beta_{1}t+\beta_{2}t^{2}+\ldots +\beta_{k}t^{k}+e_{t},$$

where *Y*_*t*_ is a time series realization of a public health indicator, *t* represents the time, and *α*, *β*_1_, …, *β*_*k*_ are parameters of the model. In case, where k = 1, the above equation becomes a simple linear regression line. The errors, *e*_*t*_, of the equation is a de-trended series and can further be modeled with an ARIMA (p,d,q) specification:

$$\nabla^{d}e_{t}=c+\phi_{1}\nabla^{d}e_{t-1}+\phi_{2}\nabla^{d}e_{t-2}+\ldots \phi_{p}\nabla^{d}e_{t-p}+\varepsilon_{t}+\theta_{1}\varepsilon_{t-1}+\theta_{2}\varepsilon_{t-2}+\ldots +\theta_{q}\varepsilon_{t-q},$$

where ∇ = difference operator (∇^1^*e*_*t*_ = *e*_*t*_-*e*_*t*-1_);

*ε*_*t*_ = white noise error term;

*c*, *ϕ*_1_,*ϕ*_2_, …, *ϕ*_*p*_, *θ*_1_, *θ*_2_, …, *θ*_*q*_ are parameters;

*p* = number of autoregressive terms;

*q* = number of moving average terms;

*d* = number of differencing.

In cases, where the errors of the trend model are white noise (no autocorrelation), no ARIMA modeling is necessary. The procedure of ARIMA model estimation is an iterative process involving four steps: model identification, parameter estimation, model validation and prediction. A number of literatures are available on computational detail of ARIMA model; however, now a day, computation can be done easily in R and other softwares as well [25–26].

## Results and discussion

### Maternal mortality ratio (MMR)

**Fig 1:**
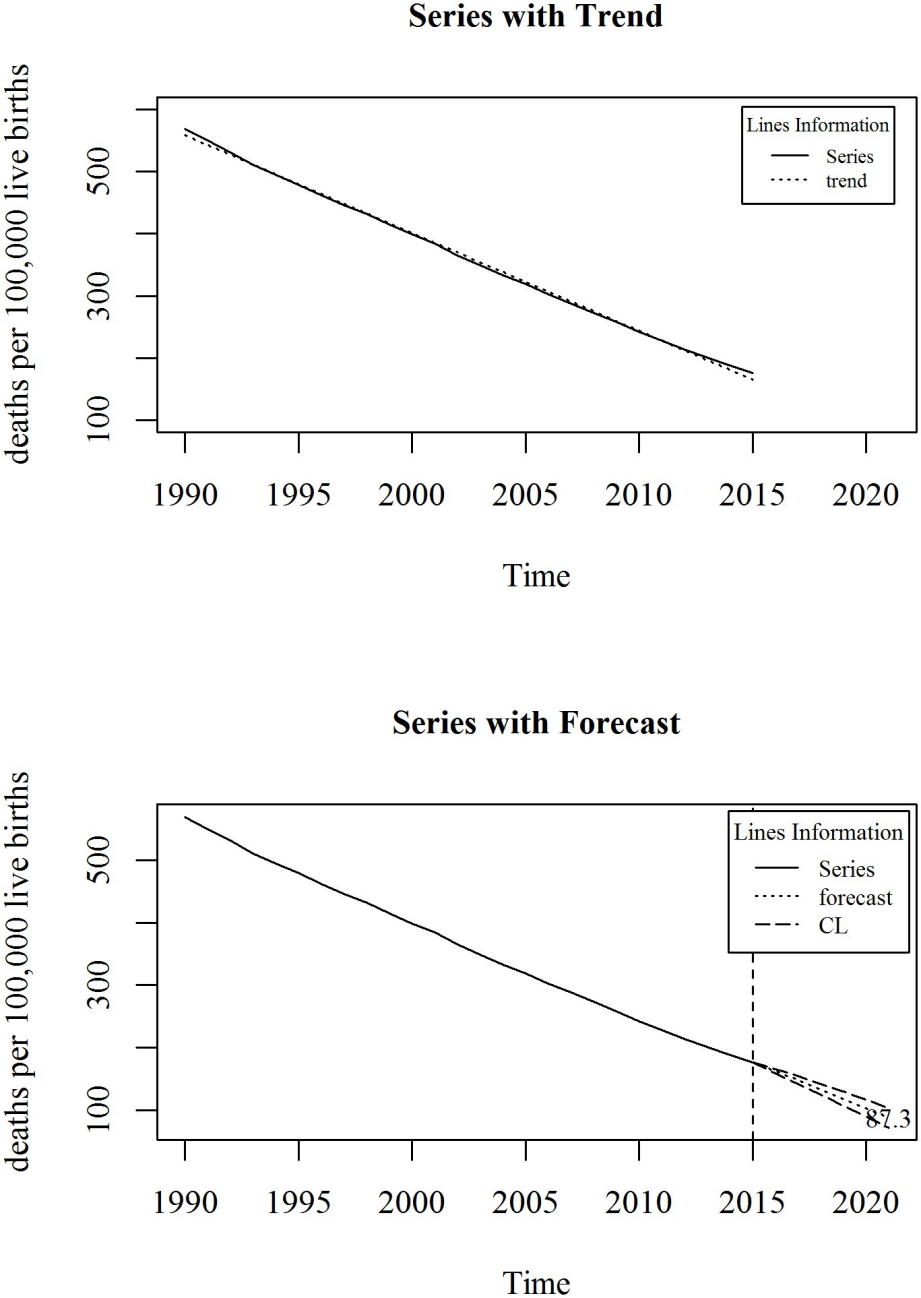
Maternal mortality ratio (MMR) in Bangladesh.

**Fig 2:**
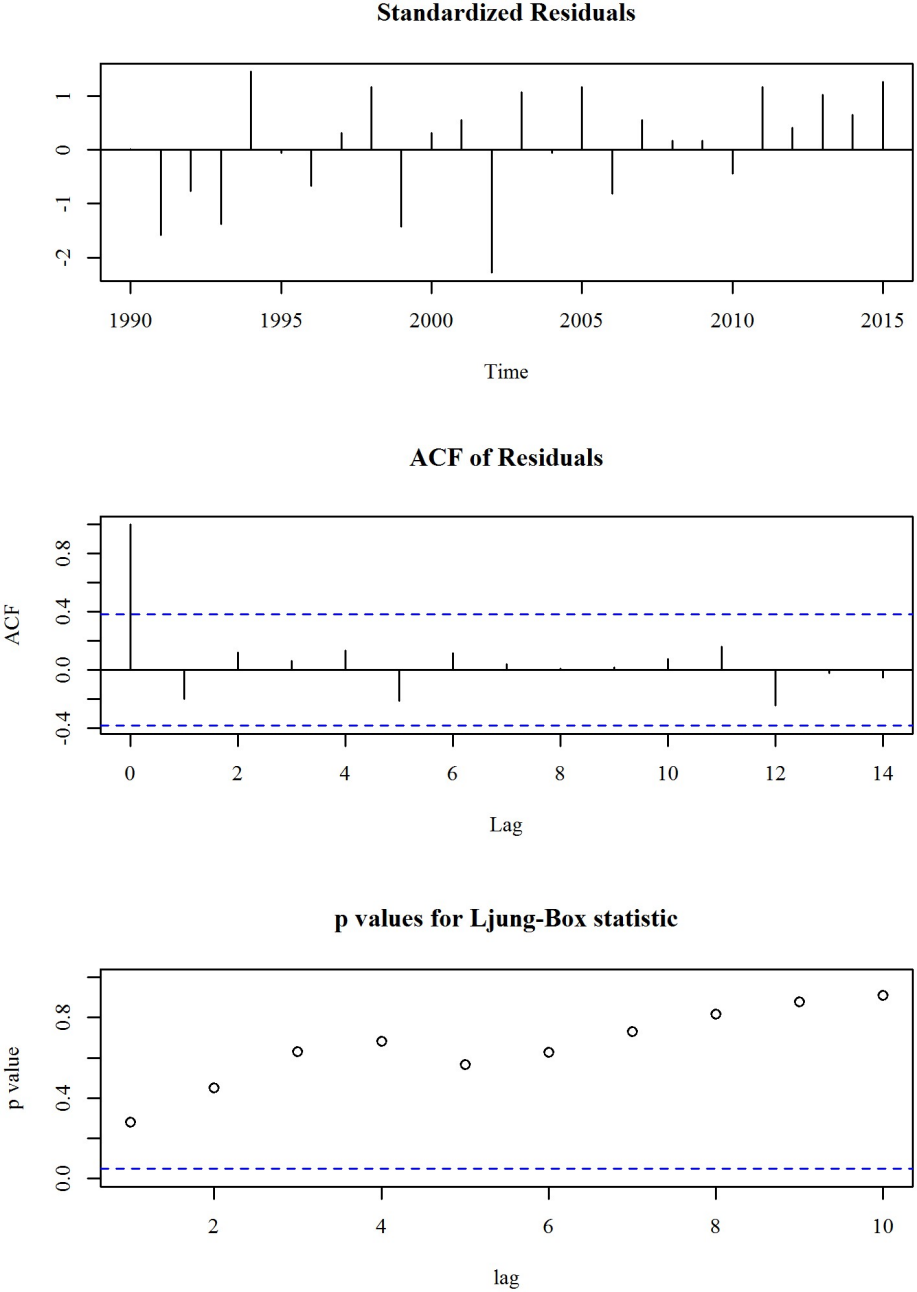
Residual diagnosis of model for maternal mortality ratio in Bangladesh.

The upper panel of Fig 1 shows the observed and trend of maternal mortality ratio (per 100,000 live births) in Bangladesh for the period 1990 to 2015. We observe a linearly decreasing trend model fits the series. A linear trend model with an ARIMA specification is given in Eqs (1) and (2). The diagnosis plot in Fig 2 shows the errors are white noise and refers that the fitted trend model and ARIMA specification is adequate. The six-year forecast is displayed in lower panel of Fig 1. It shows a declining trend of maternal mortality ratio (MMR) in Bangladesh in the following years. The predicted mortality ratio in 2021 is 87.3.

The national target for Bangladesh is to reduce maternal mortality ratio to 105 (per 100,000 live births) by 2021. We observed that maternal mortality ratio in 2015 is 176 and is predicted as 87 per in 2021. Thus, Bangladesh is in advantageous position since the maternal mortality ratio (MMR) is already lower than the national target of 2021. However, to reach maternal mortality ratio (MMR) at 70 by 2030, continuous monitoring and effective policy implication is necessary.

Trend Model:

$$Y_{t}\;=\;574.33-\;15.73t+\;e_{t}$$ (1)

p-value: 0.000 0.000

R^2^: 0.9986

ARIMA model for residuals of the trend model:

$${\nabla e}_{t}\;=\;{0.6158\nabla e_{t-1}+\varepsilon }_{t}$$ (2)

S.E: 0.1801

### Under five mortality rate

**Fig 3:**
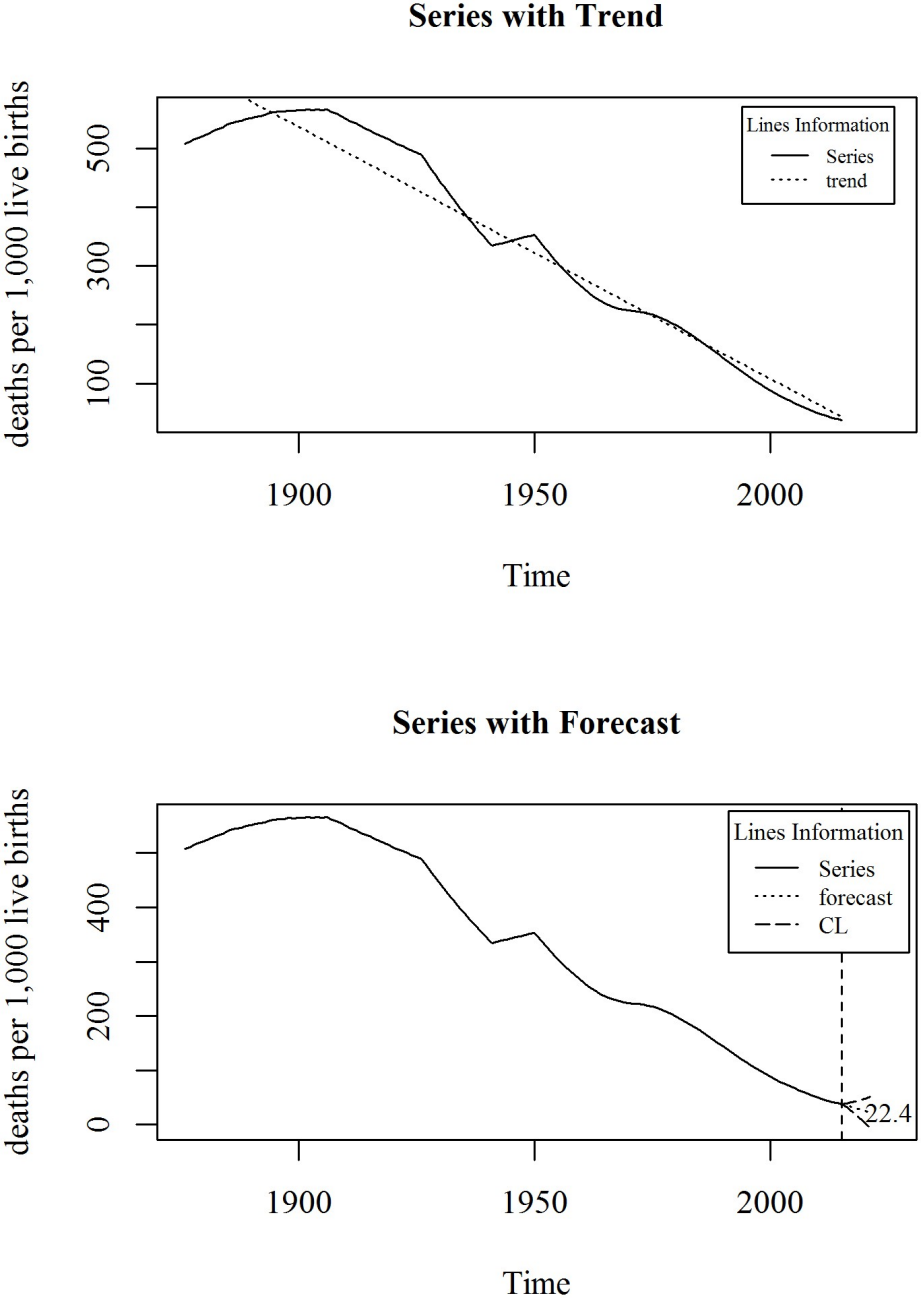
Mortality rate under five children in Bangladesh.

**Fig 4:**
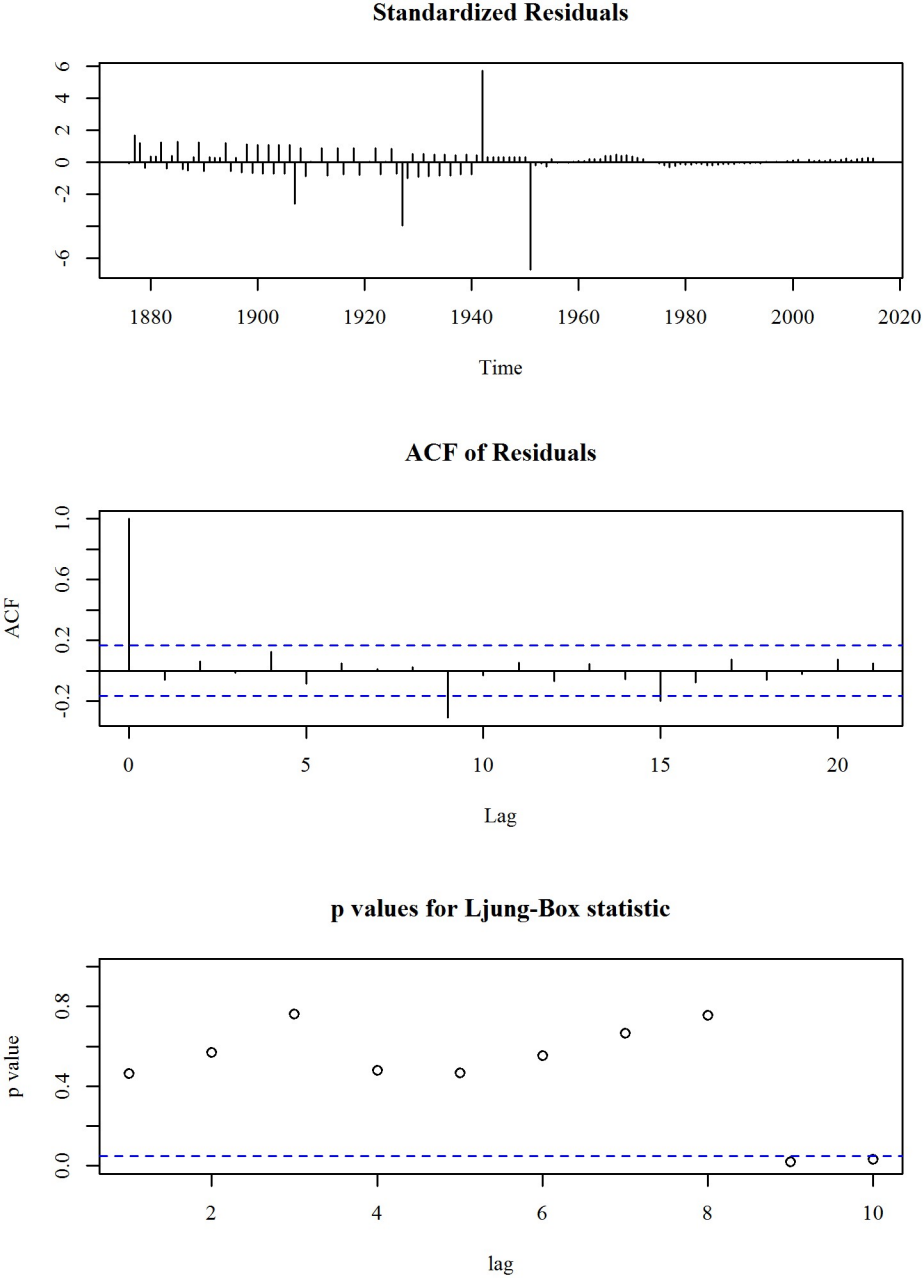
Residual diagnosis of model for mortality rate under five children in Bangladesh.

The upper panel of Fig 3 shows the observed and trend of mortality rate under-five (per 1000 live births) in Bangladesh for the period 1876 to 2015. We observe a linearly decreasing trend model fits the series. A linear trend model with an ARIMA specification is given in Eqs (3) and (4). The diagnosis plot in Fig 4 shows the errors are white noise and refers that the fitted trend model and ARIMA specification is adequate. The six-year forecast is displayed in lower panel of Fig 3. It shows a declining trend of under-five mortality in Bangladesh in the following years. The predicted under-five mortality rate in 2021 is 22.4.

By 2030, end preventable deaths of newborns and children under 5 years of age, with all countries aiming to reduce under-five mortality to at least as low as 25 per 1000 live births. The national target for Bangladesh is to reduce under-five mortality to 37 by 2021. The predicted under-five mortality rate would be 22.4 in 2021, which support the achievement of national target regarding under-five mortality rate.

Trend Model:

$$Y_{t}\;=\;641.549-4.453t+\;e_{t}$$ (3)

p-value: 0.000 0.000

R^2^: 0.9641

ARIMA model for residuals of the trend model:

$${\nabla e}_{t}\;=\;{0.9065\nabla e_{t-1}+\varepsilon }_{t}$$ (4)

S.E: 0.0363

### Neonatal mortality

**Fig 5:**
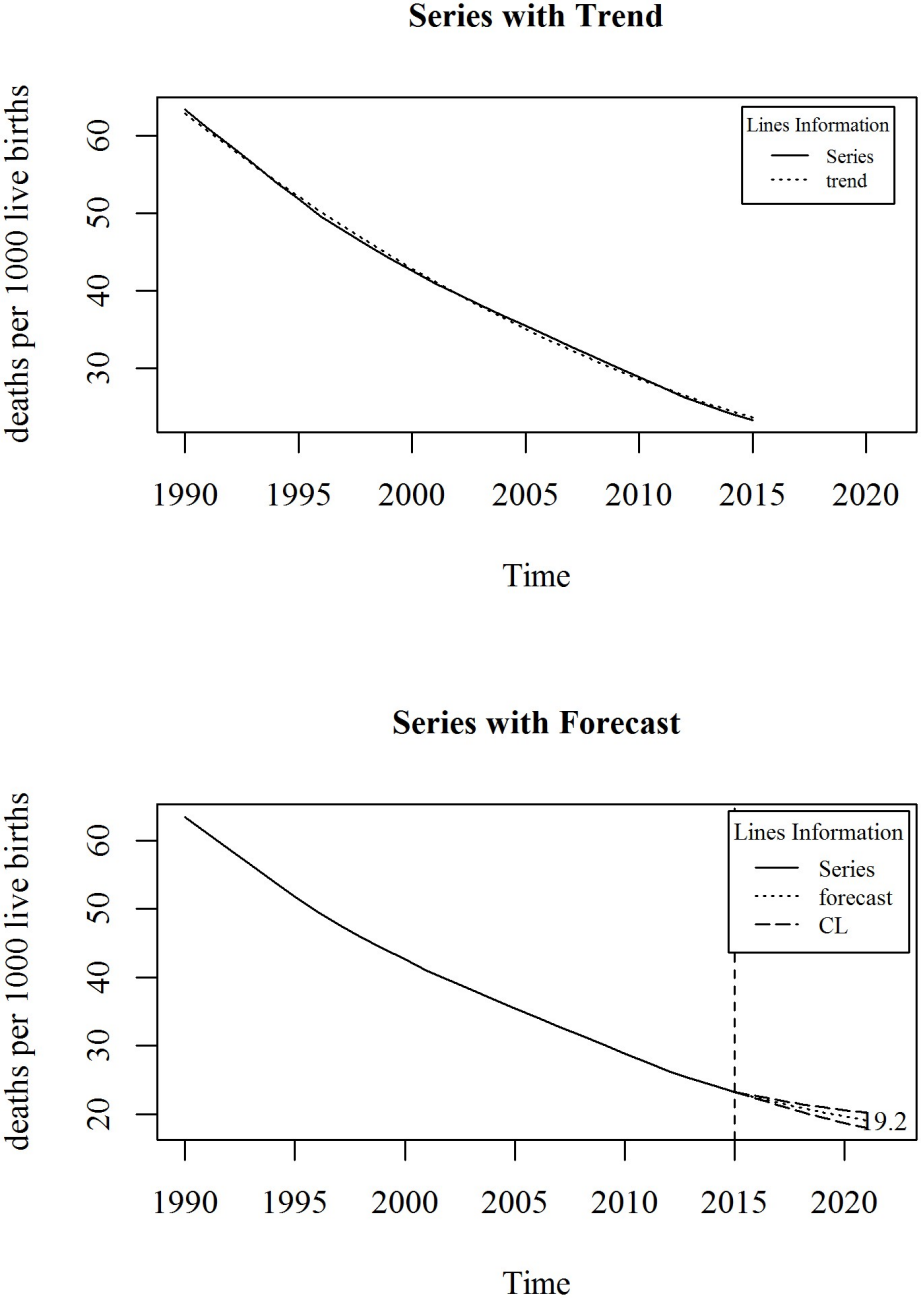
Neonatal mortality rate in Bangladesh.

**Fig 6:**
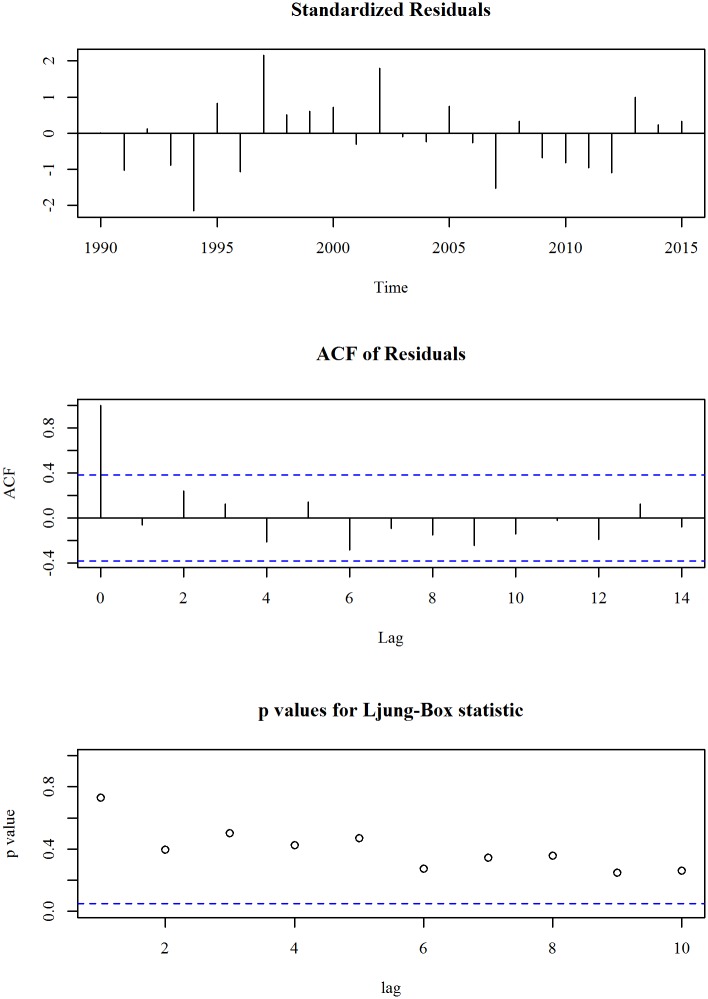
Residual diagnosis of model for neonatal mortality rate in Bangladesh.

The upper panel of Fig 5 shows the observed and trend of neonatal mortality rate (per 1000 live births) in Bangladesh for the period 1990 to 2015. We observe an overall decreasing trend in the series, however, the rate of change after 2000 is little slower than that of earlier. A two-degree polynomial trend model with an ARIMA specification is given in Eqs (5) and (6). The diagnosis plot in Fig 6 shows the errors are white noise and refers that the fitted trend model and ARIMA specification is adequate. The six-year forecast is displayed in lower panel of Fig 5. It shows a declining trend of neonatal mortality rate in Bangladesh in the following years. The predicted neonatal mortality rate in 2021 is 19.

About neonatal mortality the vision is to reduce neonatal mortality to at least as low as 12 per 1000 live births by 2030. Our country has a national target regarding neonatal mortality rate that is reduce neonatal mortality to only 21 per 1000 live births. From this study we observed a declining trend. The observed neonatal mortality rate of Bangladesh in 2015 is 23.3 and is predicted as 19 in 2021. Like as maternal mortality, monitoring and policy implication is necessary to reach the national target of 2021.

Trend Model:

$$Y_{t}\;=\;65.165\;-2.338t+\;0.02869t^{2}+e_{t}$$ (5)

p-value: 0.000 0.000 0.000

R^2^: 0.999

ARIMA model for residuals of the trend model:

$${\nabla e}_{t}\;=\;{0.783\nabla e_{t-1}+\varepsilon }_{t}$$ (6)

S.E: 0.1145

### Prevalence of stunting in under-five children

**Fig 7:**
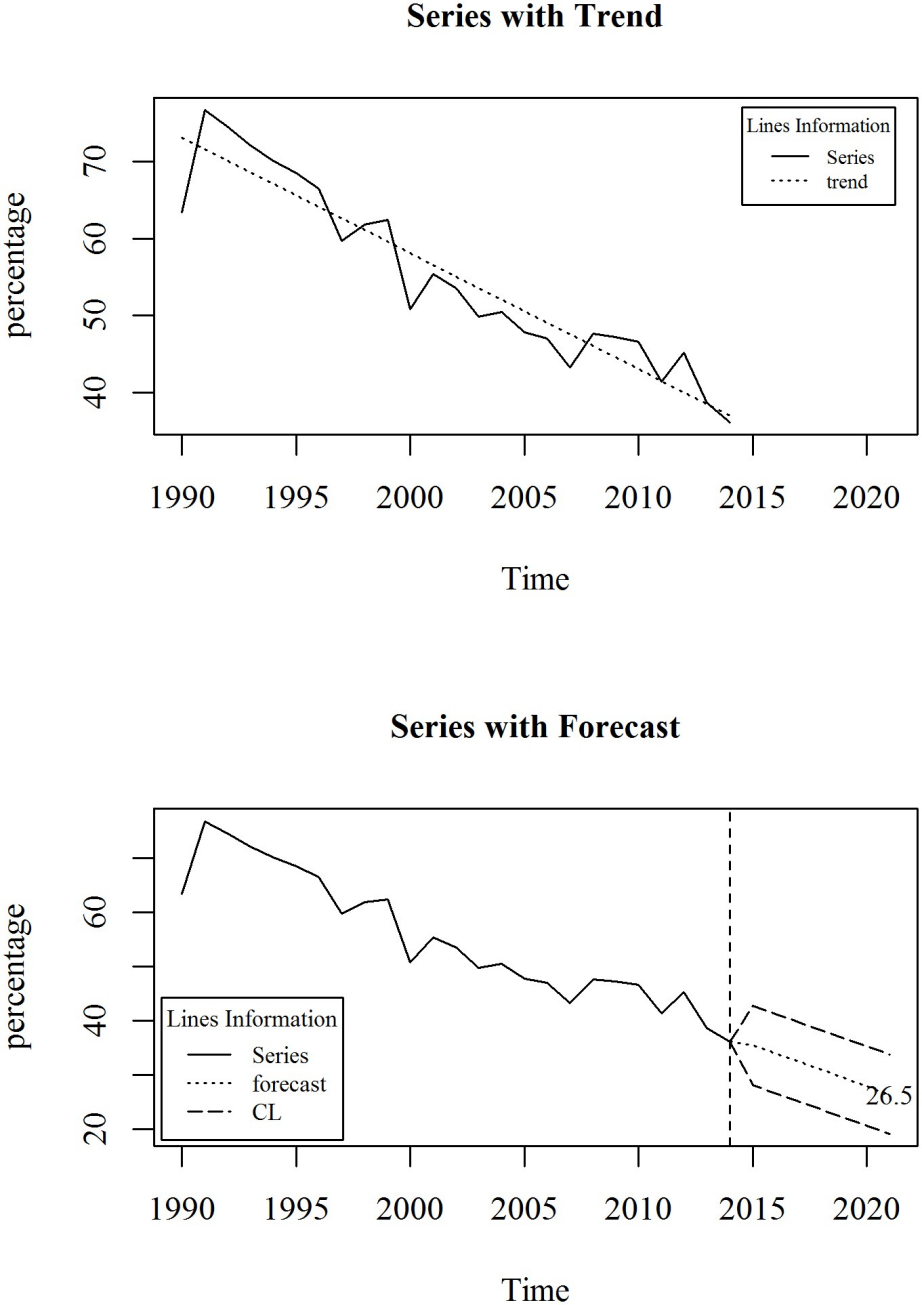
Prevalence of stunting, height for age (% of children under 5) in Bangladesh.

**Fig 8:**
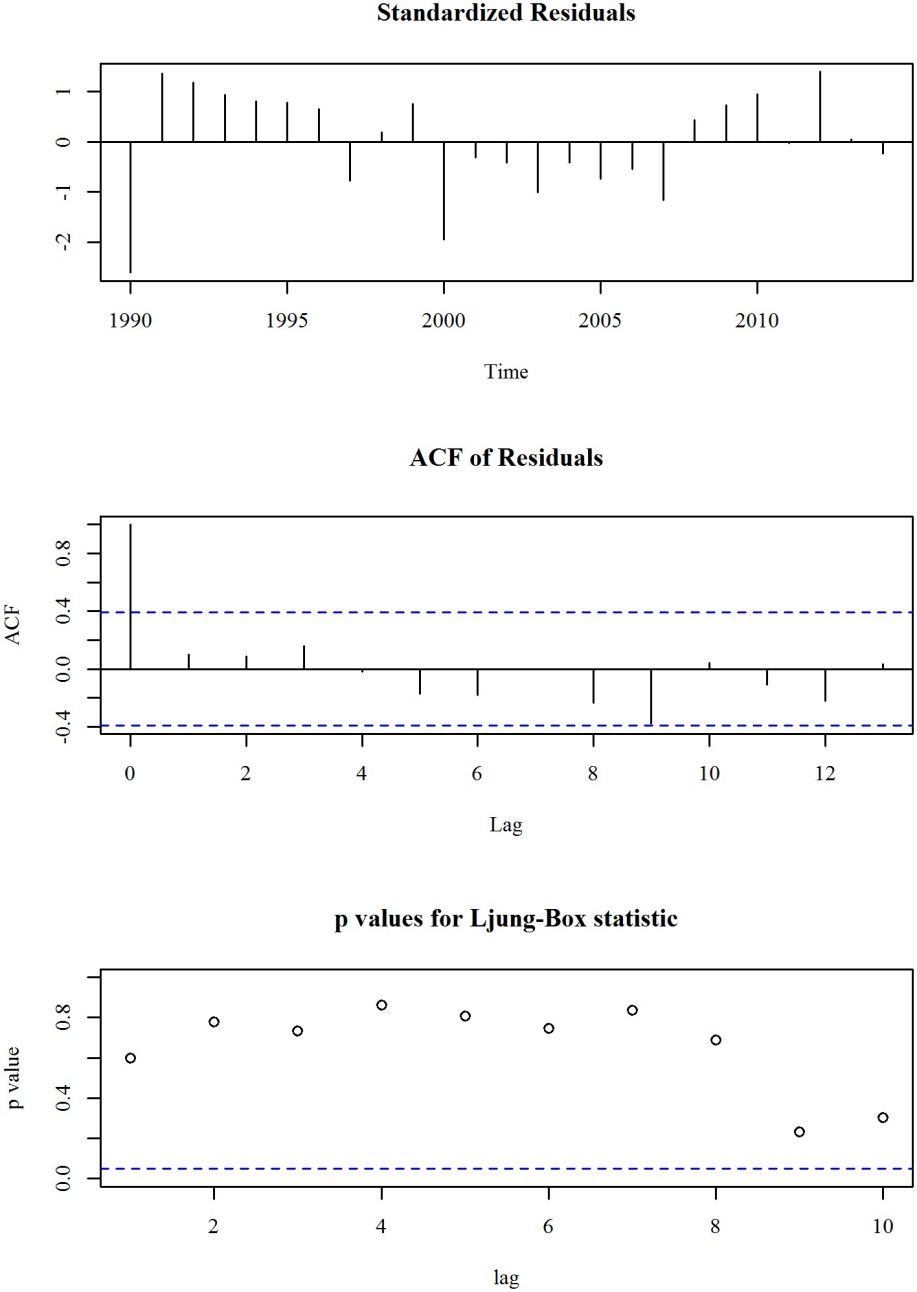
Residual diagnosis of model for prevalence of stunting in Bangladesh.

The upper panel of Fig 7 shows the observed and trend of prevalence of stunting, height for age (% of children under 5) in Bangladesh for the period 1990 to 2014. We observe a linearly decreasing trend model fits the series. A linear trend model with an ARIMA specification is given in Eqs (7) and (8). The diagnosis plot in Fig 8 shows the errors are white noise and refers that the fitted trend model and ARIMA specification is adequate. The seven-year forecast is displayed in lower panel of Fig 7. It shows a downward trend of prevalence of stunting in Bangladesh in the following years. The predicted prevalence of stunting in 2021 is 26.5.

In target 2.2 of SDG declared to end all forms of malnutrition by 2030. The health directorate of Bangladesh make national target regarding this indicator. Their target is to decline the prevalence of stunting to 25% by 2021. From this study we found that in Bangladesh by 2021, the forecasted percentage of stunting is 26.5%.

Trend Model:

$$Y_{t}\;=\;74.62-\;1.50t+\;e_{t}$$ (7)

p-value: 0.000 0.000

R^2^: 0.8948

ARIMA model for residuals of the trend model:

$$e_{t}\;=\;\varepsilon_{t\;}$$ (8)

### Prevalence of wasting in under-five children

**Fig 9:**
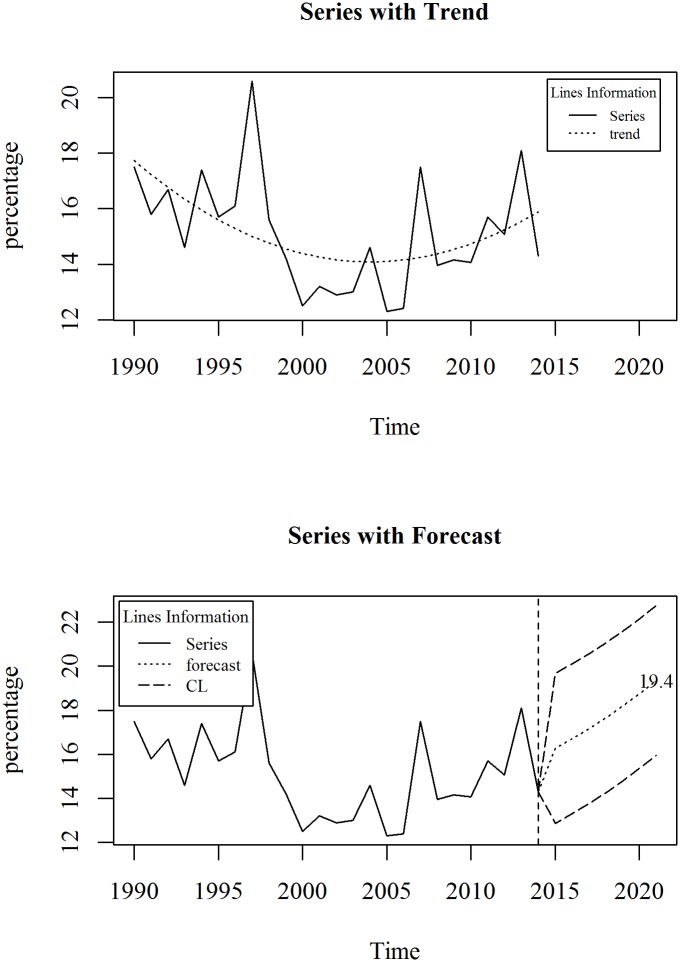
Prevalence of wasting, weight for height (% of children under 5) in Bangladesh.

**Fig 10:**
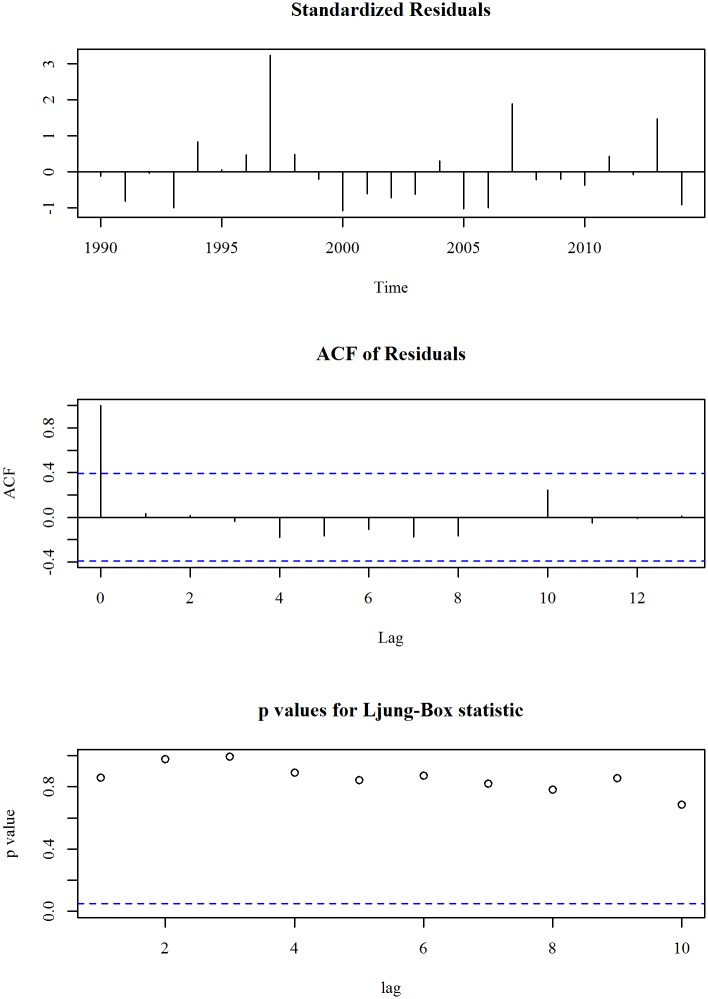
Forecast of prevalence of wasting (% of children under 5) in Bangladesh.

The upper panel of Fig 9 shows the observed and trend of prevalence of wasting, weight for height (% of children under 5) in Bangladesh for the period 1990 to 2014. We observe that the series is oscillating over time refers non-linearity. Though decreasing trend is observed until 2005, an increasing trend in prevalence of wasting is observed after that. A second degree polynomial trend model with an ARIMA specification is given in Eqs (9) and (10). The diagnosis plot in Fig 10 shows the errors are white noise and refers that the fitted trend model and ARIMA specification is adequate. The seven-year forecast is displayed in lower panel of Fig 9. It shows an increasing trend of prevalence of wasting in Bangladesh in the following years. The predicted prevalence of wasting in 2021 is 19.4.

Like as stunting, the health directorate of Bangladesh make national target regarding this indicator. Their target is to decline the prevalence of wasting to 25% by 2021. From this study we found that in Bangladesh by 2021, the forecasted percentage of wasting is 19.4%.

Trend Model:

$$Y_{t}\;=\;18.27-\;0.56t+0.018t^{2}+\;e_{t}$$ (9)

p-value: 0.000 0.01 0.03

R^2^: 0.2567

ARIMA model for residuals of the trend model:

$$e_{t}\;=\;\varepsilon_{t\;}$$ (10)

## Conclusion

Bangladesh is one of the developing countries of the world. Child and maternal health and nutrition-related indicators has been improved over past few decades. The study summarizes that maternal mortality ratio (MMR), under-five mortality rate and neonatal mortality rate shows declining trend in Bangladesh. The current trend and future prediction of these indicators considered in this study support satisfactory development and on a track to reach national target of Bangladesh 2021. Further, more initiatives need to achieve related SDGs 2030.

In spite of gaining a remarkable change in different public health indicators in Bangladesh, malnutrition is still a concerning issue for us. This study observed a decreasing trend in prevalence of stunting in Bangladesh, while and prevalence of wasting in recent years is increasing. The predicted percentage of stunting is higher than the national target-2021. Eventually if we do not take necessary steps, we will not be able to reach SDG target 2.2 about malnutrition. Assuring a sound maternal and child health of a country is essential for sound public health status of the country. Reductions in stunting and other forms of under nutrition can be achieved through proven interventions which include improving women’s nutrition, especially before, during and after pregnancy; early and exclusive breastfeeding; timely, safe, appropriate and high-quality complementary food; and appropriate micronutrient interventions. Progress on child and maternal nutrition is needed to reduce high levels of under nutrition on child survival, growth and development.

In this study, maternal and child health indictors are analyzed. In further study, other health indicators like communicable and non-communicable diseases with their dependence with improved water and hygiene and sanitation could be analyzed using time series tools and techniques. We left these topics for future research.

## Supporting information

S1 Table Data maternal and child mortality in Bangladesh. (XLSX)
